# Or47b plays a role in *Drosophila* males' preference for younger mates

**DOI:** 10.1098/rsob.160086

**Published:** 2016-06-08

**Authors:** Luming Zhuang, Ying Sun, Mi Hu, Chenxi Wu, Xiaojin La, Xinhong Chen, Yu Feng, Xingjun Wang, Yujia Hu, Lei Xue

**Affiliations:** 1 Department of Interventional Radiology, Shanghai 10th People's Hospital, Shanghai Key Laboratory of Signaling and Disease Research, School of Life Science and Technology, Tongji University, Shanghai 200092, People's Republic of China; 2 Kent School, 1 Macedonia Rd, Kent, CT 06757, USA; 3 College of Chinese Medicine, North China University of Science and Technology, Tangshan 063000, People's Republic of China

**Keywords:** Or47b, *Drosophila*, courtship preference

## Abstract

Reproductive behaviour is important for animals to keep their species existing on Earth. A key question is how to generate more and healthier progenies by choosing optimal mates. In *Drosophila melanogaster*, males use multiple sensory cues, including vision, olfaction and gustation, to achieve reproductive success. These sensory inputs are important, yet not all these different modalities are simultaneously required for courtship behaviour to occur. Moreover, the roles of these sensory inputs for male courtship choice remain largely unknown. Here, we demonstrate that males court younger females with greater preference and that olfactory inputs are indispensable for this male courtship choice. Specifically, the olfactory receptor Or47b is required for males to discriminate younger female mates from older ones. In combination with our previous work indicating that gustatory perception is necessary for this preference behaviour, our current study demonstrates the requirement of both olfaction and gustation in *Drosophila* males' courtship preference, thus providing new insights into the role of sensory cues in reproductive behaviour and success.

## 1. Introduction

In *Drosophila melanogaster*, males adopt various ways of sensory inputs to judge whether a subject (usually a *Drosophila* female) is worth courting. These sensory cues are visual, olfactory and gustatory, which are implemented via peripheral sensory organs including eyes, antennae, maxillary palp, labella, leg tarsi, etc. [1]. While vision helps male flies to find and track the females, olfactory receptor neurons (ORNs) and gustatory receptor neurons (GRNs) harboured in the olfactory and gustatory appendages assist male flies in detecting pheromones or other chemical compounds to further confirm the availability of female flies [2].

In the initiation of courtship, male flies use vision to track and keep up with the pace of female flies (if the female flies are moving), which is termed as following behaviour [3]. Nevertheless, the following behaviour is seldom observed in the courtships of males with impaired vision [3–5]. Despite the fact that males can still court in darkness (without visual inputs), the involvement of vision allows males to court females more accurately [6].

In addition to visual cues, male flies also take advantage of olfaction to achieve an effective courtship. The olfactory information is received by two major appendages that are called the third antennal segments and the maxillary palps, respectively [7,8]. The ORNs are located in the sensilla housed in the two aforementioned appendages [8]. Among all ORNs, Or67d neurons are reported to be pheromone perception ORNs [9]. In addition, a recently published study confirmed that Or47b neurons also participate in pheromone perception [10]. Both Or67d and Or47b in males express male-specific fruitless protein (Fru^M^) encoded by P1 promoter transcripts of the *fruitless* gene. Fru^M^ is reported to be necessary and sufficient to elicit male courtship behaviour [11–13]. Another group of chemosensory neurons named ionotropic receptor 84a (IR84a) neurons, which belong to the ionotropic glutamate receptor family, also express Fru^M^ [14]. However, the ligand of this receptor, phenylacetic acid, is not considered as a sex pheromone in flies, but rather an acid that is rich in fruits. Or67d neurons target the DA1 glomerulus, whereas Or47b neurons target the VA1v glomerulus, which are much larger in males than in females; besides, disrupting the activity of these Fru^M^-positive neurons impairs male courtship behaviour [11]. This evidence implies that these two groups of ORNs are components of male-specific behaviour.

In the tapping and licking steps of the courtship behaviour, males use gustatory cues to detect contact pheromones and compare their potential mates. The gustatory perception and its neural circuitry have been intensively studied, and mutations on several gustatory receptors (GRs) result in less male courtship to females, attesting the importance of gustatory perception in courtship behaviour [15].

Our previous work focusing on *Drosophila* male courtship preference demonstrated that males could discriminate younger females and older ones, and made more efforts to court younger ones [16]. Besides, some of our data unravelled the role of gustatory perception in male courtship preference behaviour [17]. Here, we investigate the possible function of visual and olfactory sensory inputs in male courtship preference behaviour by sequential analysis, and establish new parameters for assessing male courtship preference. We show that males' preference for younger mates was entirely eliminated when an olfactory defect was present, but remained unaffected by impaired visual inputs. Furthermore, the olfactory receptor Or47b and Or47b-expressing neurons are required for males' preference for younger mates.

## 2. Material and methods

### 2.1. *Drosophila* strains and rearing

*Oregon R* (BL# 5), *ninaB^1^* (BL# 24776), *Or67d-Gal4* (BL# 9998) and *Or47b-Gal4* (BL# 9983) were obtained from Bloomington *Drosophila* Stock Center; *UAS-hid* was a gift from Dr John R. Nambu; *Or83b-Gal4* was a gift from Dr Zuoren Wang and *Or47b^+/+^*, *Or47b^2/2^* and *Or47b^3/3^* were generously provided by Dr Liming Wang. Flies were maintained on a standard corn flour, yeast and agar medium under a 12 L : 12 D cycle at 25°C. Naive male and virgin female flies were collected at eclosion. Males were kept individually for 3 days before behavioural tests.

### 2.2. Behavioural assays

**Figure 1.**
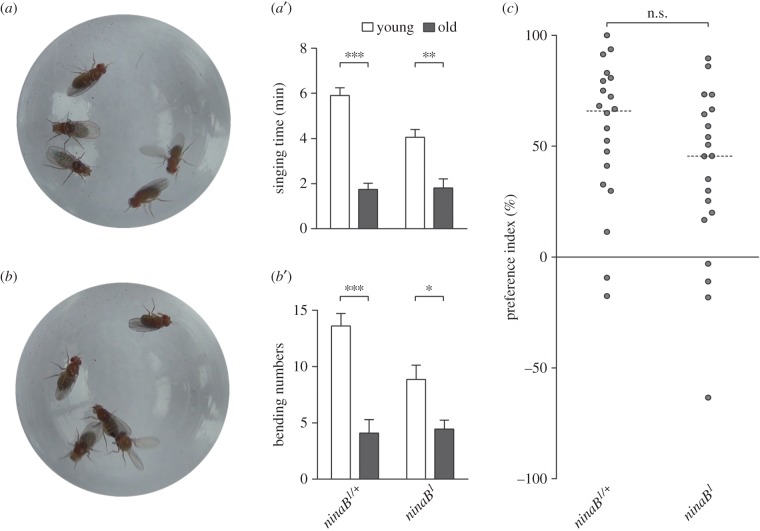
Males still prefer younger mates when their visual inputs are impaired. (*a*,*a*’) Singing time of control males (*+/ninaB^1^*) and *ninaB^1^* mutant males (*ninaB^1^*) towards younger virgin females (bars coloured white) and older ones (bars coloured grey) in courtship choice assays. Mean ± s.e.m. The sample volume (*n*) is 20 for all the genotypes in choice assays throughout this study except where indicated separately. **p < 0.01, ***p < 0.001, related-samples Wilcoxon signed-rank test. (*b*,*b*’) Bending numbers of control males and *ninaB^1^* mutant males towards younger virgin females (white bars) and older ones (grey bars) in courtship choice assays. Mean ± s.e.m., *p < 0.05, ***p < 0.001, related-samples Wilcoxon signed-rank test. (*c*) Preference indices of control males and *ninaB^1^* mutant males in courtship choice assays. Scatter dot plot with dotted line at median. n.s., *p* > 0.05, Mann–Whitney *U*-test.

Courtship choice assays were performed as described before [16,17]. Briefly, in choice assays, a naive male (3 days after eclosion) was paired with two younger virgin females (3 days after eclosion) and two older ones (30 days after eclosion). We used eye colour to distinguish younger females and older ones (e.g. red eye females as younger ones while white eye females as older ones, or vice versa.). All tests were recorded for 10 min with an HDR-CX270 digital video camera (Sony). After recording, videos were analysed by a researcher blind to the genotypes of males or age markers of females, using Noldus EthoVision XT software (Noldus Information Technology). The singing time (ST) is the time that males displayed one wing vibration (figure 1*a*) during the observing time (10 min) in a courtship choice assay, and the bending numbers (BN) are the numbers of times that males bent their abdomens (figure 1*b*) during the observing period. Therefore, the STs of a male towards younger virgin females and older ones are abbreviated as ST_Y_ and ST_O_, respectively. Similarly, the BNs of this male towards younger virgin females and older ones are BN_Y_ and BN_O_, respectively. The relative difference between ST_Y_ and ST_O_ is defined as ΔST: ΔST = (ST_Y_ − ST_O_)/(ST_Y_ + ST_O_). Similarly, the relative difference between BN_Y_ and BN_O_ is ΔBN: ΔBN = (BN_Y_ − BN_O_)/(BN_Y_ + BN_O_). In this study, we defined the preference index (PI) as the arithmetic mean of the relative difference between ST_Y_ and ST_O_ and that between BN_Y_ and BN_O_: PI = (ΔST + ΔBN)/2.

For the copulation assays, we paired a naive male (3 days after eclosion) together with two younger virgin females (3 days after eclosion) and two older ones (30 days after eclosion). We still used eye colour to distinguish younger females from older ones. All tests were recorded for 30 min and analysed by a researcher blind to the genotypes of males or age markers of females. The mating rate was calculated as the percentage of the number of younger or older females successfully mated within 30 min versus the total number of females.

To examine females' egg laying and offspring viability, younger (3 days after eclosion) or older females (30 days after eclosion) were single-paired with wild-type males (3 days after eclosion) in vials containing standard food and were observed for 30 min. Once a successful copulation occurred, the males were removed and the females were transferred to new vials containing standard food every 24 h, and the number of eggs in each vial was counted. The number of eclosed flies was counted from the 9th day after females' copulation, and the eclosion rate was calculated as the percentage of eclosion number versus total egg number in a single vial.

### 2.3. Statistical analysis

As the ST and BN were generally not normally distributed and the sample volumes were relatively small, non-parametric tests were employed in statistical analysis. Comparisons of intragroup STs (ST_Y_ and ST_O_) and BNs (BN_Y_ and BN_O_) in courtship choice assays used the related-samples Wilcoxon signed-rank test. PIs in choice assays were compared using the Mann–Whitney *U*-test or the Kruskal–Wallis test followed by Dunn's post hoc, according to different numbers of groups. For the egg-laying assays, parametric tests were applied according to the normal distribution of the samples.

## 3. Results and discussion

*Drosophila* male courtship behaviour is a stereotyped ritual consisting of several steps including orientation, tapping, singing, licking and attempted copulation [18,19]. During these steps, a male fly uses multiple sensory cues to determine its potential mates. Upon finding the target is promising, the male fly will vibrate one of its two wings (figure 1*a*) to produce a courtship-specific sound. Additionally, it will also bend its abdomen (figure 1*b*) in an attempt to copulate with the courting subject [20–22]. Both of these actions are clear and easy to observe; in addition, both of them robustly manifest the males' courting wishes, and thus can be used to set parameters assessing males' courtship preference. Therefore, we employed the amount of time males sing to younger/older females (designated as ST_Y_/ST_O_, respectively) and the numbers of times they bend their abdomens to younger/older females (BN_Y_/BN_O_) as parameters of males' courting wishes. In order to quantify the males' courtship preference, we combined these two parameters together to define a PI (see Material and methods for details).

In the process of courtship, visual information plays a key role and is important for males to establish and maintain the contact with females [6]. To investigate the possible functions of visual inputs in males' courtship preference behaviour, two different experimental approaches were undertaken: one is using *ninaB^1^* mutant males and performing the courtship choice assays under white light, and the other is using wild-type (*Oregon R*) males and performing the courtship choice assays under dim red light, which equals darkness to flies [23]. The *ninaB^1^* mutant males have a grave defect in vision because NinaB is a vital ingredient for visual pigment production and *ninaB* mutation blocks the synthesis of the rhodopsin chromophore retinal [24]. In courtship choice assays, the *ninaB^1^* males spent significantly more time 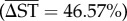 singing to younger females than to older ones (figure 1*a*’) and they also bent their abdomens more frequently 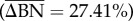 to younger females than to older ones (figure 1*b*’). In addition, the PI of *ninaB^1^* males was not significantly different from that of the control group (*ninaB^1^*/+; figure 1*c*). Consistent with these data, when the courtship choice assays were performed under dim red light, wild-type males still discriminated younger females from older ones (electronic supplementary material, figure S1A,A’,B,B’), as they did under white light (electronic supplementary material, figure S1A,A’,B,B’). The PI of wild-type males under white light showed no significant difference from that under dim red lights (electronic supplementary material, figure S1C). Taken together, these results indicate that males with impaired visual sensory inputs still distinguish younger females from older ones.

With such strong courtship preference for younger mates, it is possible that males can get reproductive benefits. To investigate this possibility, we first examined the number of eggs laid by younger or older females after copulation with 3-day-old wild-type males. We found that on post-copulation days 2, 3 and 4, the number of eggs laid by younger females was significantly greater than that by older ones (electronic supplementary material, figure S2A), and the total egg-laying number of younger females was also dramatically greater than that of older ones (electronic supplementary material, figure S2A’). We also checked the eclosion rates of offspring produced by both younger and older females. Flies started to eclose on post-copulation day 9 and reached a maximum on day 10 (electronic supplementary material, figure S2B). Although the eclosion rates varied between younger and older females on each day, the overall eclosion rates were comparable between the two groups (electronic supplementary material, figure S2B’). These results suggest that younger females can produce more progenies than older ones, which is a good reason for males to choose younger mates.

**Figure 2.**
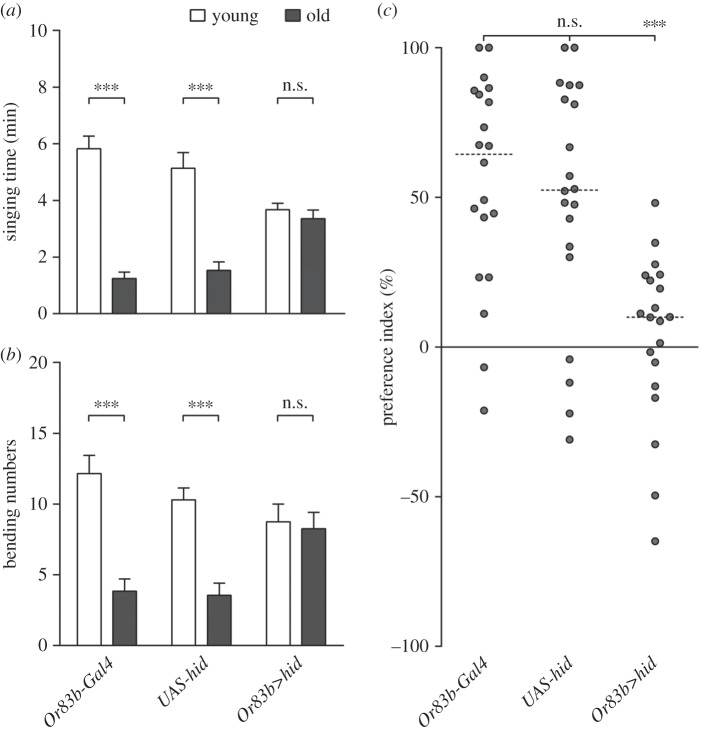
Olfactory inputs play a role in males' preference behaviour. (*a*) Singing time of *Or83b-Gal4* males (*Or83b-Gal4/+*), *Or83b*>*hid* males (*Or83b-Gal4/UAS-hid*) and *UAS-hid* males (*+/UAS-hid*) towards younger virgin females (white) and older ones (grey) in courtship choice assays. Mean ± s.e.m. n.s., *p* > 0.05, ***p < 0.001, related-samples Wilcoxon signed-rank test. (*b*) Bending numbers of *Or83b-Gal4* males, *Or83b*>*hid* males and *UAS-hid* males towards younger virgin females (white) and older ones (grey) in courtship choice assays. Mean ± s.e.m. n.s., *p* > 0.05, ***p < 0.001, related-samples Wilcoxon signed-rank test. (*c*) Preference indices of *Or83b-Gal4* males, *Or83b*>hid males and *UAS-hid* males in courtship choice assays. Scatter dot plot with dotted line at median. n.s., *p* > 0.05, ***p < 0.001, Kruskal–Wallis test, Dunn's post hoc.

*Drosophila*, like other insects, use sex pheromones to coordinate their reproductive behaviours. As female-specific volatile pheromones are detected by males' olfaction in the courtship behaviour [25], we examined the potential role of olfactory inputs in males' preference for younger females. Most *Drosophila* olfactory sensory neurons express an olfactory receptor gene *Or83b*, which is highly conserved between insect species [26]. Or83b does not bind odorant ligand but serves as a protein chaperone forming a complex with other regular olfactory receptors to mediate olfactory perception [26]. Thus, we used the proapoptotic gene *head involution defective* (*hid*) [27] to ablate males' Or83b neurons (*Or83b-Gal4*/*UAS-hid*) so that these males had gross olfactory deficiency. We introduced these Or83b neuron-ablated males into courtship assays and observed that these males spent almost the same time in singing 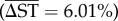 to younger females and to older ones (figure 2*a*). They also exhibited the equivalent frequency in bending their abdomens 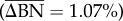 to younger females and to older ones (figure 2*b*). As a result, the PI of these males was conspicuously lower than that of corresponding control males (*Or83b-Gal4*/+ or +/*UAS-hid*) (figure 2*c*). Thus, malfunction of olfactory sensory neurons abrogates males' ability to differentiate younger females from older ones, suggesting olfactory sensory cues greatly facilitate the males' preference for younger mates.

**Figure 3.**
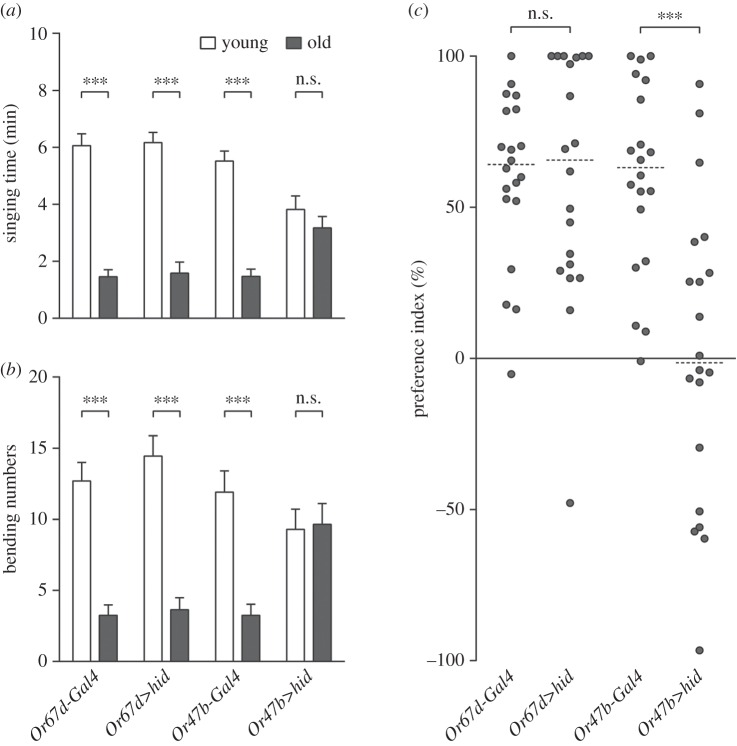
Ablating Or47b neurons disrupts males' preference for younger mates. (*a*) Singing time of *Or67d-Gal4* males (*Or67d-Gal4/+*), *Or67b*>*hid* males (*Or67d-Gal4/UAS-hid*), *Or47b-Gal4* males (*Or47b-Gal4/+*) and *Or47b*>*hid* males (*Or47b-Gal4/UAS-hid*) towards younger virgin females (white) and older ones (grey) in courtship choice assays. Mean ± s.e.m. n.s., *p* > 0.05, ***p < 0.001, related-samples Wilcoxon signed-rank test. (*b*) Bending numbers of *Or67d-Gal4* males, *Or67b*>*hid* males, *Or47b-Gal4* males and *Or47b*>*hid* males towards younger virgin females (white) and older ones (grey) in courtship choice assays. Mean ± s.e.m. n.s., *p* > 0.05, ***p < 0.001, related-samples Wilcoxon signed-rank test. (*c*) Preference indices of *Or67d-Gal4* males, *Or67b*>*hid* males, *Or47b-Gal4* males and *Or47b*>*hid* males in courtship choice assays. Scatter dot plot with dotted line at median. n.s., *p* > 0.05, ***p < 0.001, Mann–Whitney *U*-test.

Next, we sought to identify which set of ORNs was responsible for the discriminating ability of male flies. As Or67d neurons and Or47b neurons are reported as pheromone-sensing ORNs [9–11], we separately ablated the activity of these two types of neurons by using *UAS-hid* (*Or67d-Gal4*/*UAS-hid* or *Or47b-Gal4*/*UAS-hid*). The Or67d neuron-ablated males were still willing to invest more time singing 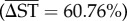 to younger females than to older ones (figure 3*a*). Additionally, they were more vigorous in attempting to copulate 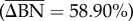 with younger females than with older ones (figure 3*b*). Moreover, the PI of Or67d neuron-ablated males showed no discrepancy to that of the control group (*Or67d-Gal4*/+; figure 3*c*). These data suggest that dysfunction of Or67d neurons does not perturb males' preference for younger mates. On the other hand, the Or47b neuron-ablated males spent similar amounts of time singing to younger females 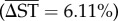 and to older ones (figure 3*a*). The amount of times they tried to copulate with younger females 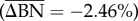 also resembled that of older ones (figure 3*b*). The PI of Or47b neuron-ablated males drastically decreased compared with that of control males (*Or47b-Gal4*/+) (figure 3*c*), illustrating that loss of Or47b neurons causes males to forfeit their preference for younger females.

**Figure 4.**
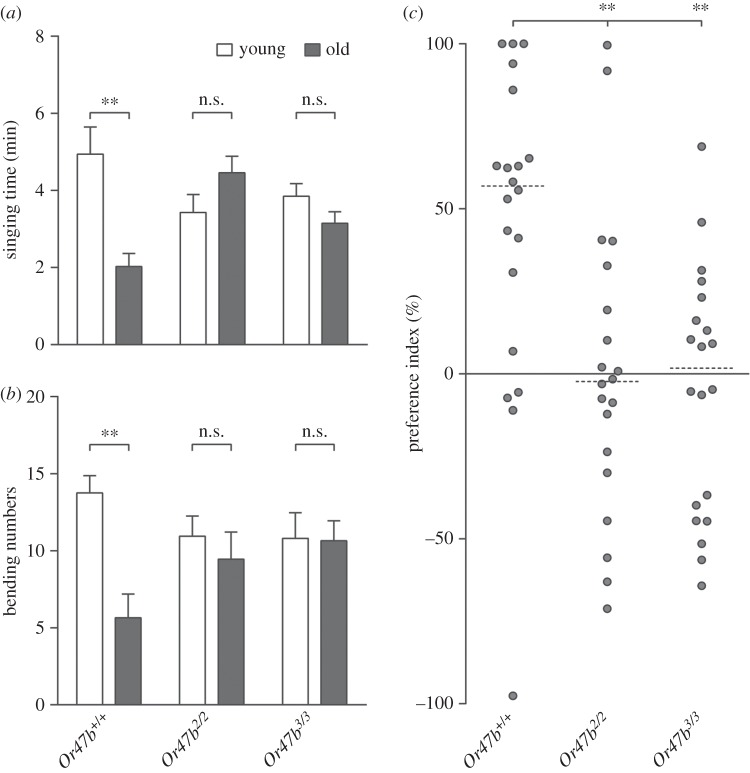
Or47b is required in males' preference behaviour. (*a*) Singing time of *Or47b^+/+^* males (*Or47b^+/+^*), *Or47b^2/2^* males (*Or47b^2/2^*) and *Or47b^3/3^* males (*Or47b^3/3^*) towards younger virgin females (white) and older ones (grey) in courtship choice assays. Mean ± s.e.m. n.s., *p* > 0.05, **p < 0.01, related-samples Wilcoxon signed-rank test. (*b*) Bending numbers of *Or47b^+/+^* males, *Or47b^2/2^* males and *Or47b^3/3^* males towards younger virgin females (white) and older ones (grey) in courtship choice assays. Mean ± s.e.m. n.s., *p* > 0.05, **p < 0.01, related-samples Wilcoxon signed-rank test. (*c*) Preference indices of *Or47b^+/+^* males, *Or47b^2/2^* males and *Or47b^3/3^* males in courtship choice assays. Scatter dot plot with dotted line at median. **p < 0.01, Kruskal–Wallis test, Dunn's post hoc.

The foregoing observation suggested the critical function of Or47b neurons in males' preference for younger mates. Owing to the broad expression of Or47b in Or47b neurons [28], we then asked whether Or47b is essential for males' courtship preference. We applied two independent *Or47b* null alleles (*Or47b^2/2^* and *Or47b^3/3^*) and their corresponding wild-type control (*Or47b^+/+^*) [29], and found that both *Or47b^2/2^* and *Or47b^3/3^* males tremendously reduced their ST (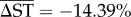 for *Or47b^2/2^* and 9.35% for *Or47b^3/3^*, respectively) to younger females (figure 4*a*). Moreover, they were less ambitious in attempting to copulate (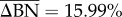 for *Or47b^2/2^* and −19.37% for *Or47b^3/3^*, respectively) with younger females (figure 4*b*). Consistently, the PIs of both *Or47b^2/2^* and *Or47b^3/3^* males severely declined, as compared with the control *Or47b^+/+^* males (figure 4*c*). We also conducted courtship choice assays on *Or47b* mutant males under dim red light. These males, as under white light, also failed to distinguish between younger and older females under dim red light (electronic supplementary material, figure S3A,B). As a result, the PIs of *Or47b* mutant males were significantly lower than that of control males (*Or47b*^+/+^; electronic supplementary material, figure S3C). These data demonstrate that with the loss of Or47b function, males become less proficient at discerning younger females from older ones. Hence, we conclude that Or47b is required for males' preference for younger mates.

So far, we had demonstrated the essential role of Or47b in males' courtship preference. However, whether males' courtship preference leads to copulation success remained unresolved. Therefore, we used these *Or47b* mutant males as a loss of function tool to study this question. We checked the copulation success rates of both *Or47b*^*2*/*2*^ and *Or47b*^*3*/*3*^ mutant males and *Or47b*^+/+^ control ones. In copulation experiments, both control and mutant males had higher copulation success rates with younger females than with older ones (electronic supplementary material, figure S4), suggesting that males' courtship preference was not necessarily a sign of copulation success. Among animals, a successful copulation is consensual. In this way, the copulation success can also be greatly influenced by the females' copulation decisions, which makes it possible that females' copulation decisions would thus have an effect on males' courtship preference. To investigate this possibility, we used decapitated females (without interactions or feedbacks with males) as courting subject for males in courtship choice assays. Faced with decapitated females, wild-type males still strongly preferred younger females to older ones (electronic supplementary material, figure S5). Thus, females' mating decisions or their feedbacks during the interaction with courting males have no effect on males' courtship preference.

Throughout the entire lifespan of *Drosophila* males, courtship is crucial and basic. Choosing the right mate benefits males with more abundant and genetically better offspring. In courtship, males integrate visual, olfactory and gustatory information to start and maintain the courtship process, and therefore these sensory inputs are all important for this behaviour [23]. Nevertheless, several lines of evidence suggest that even in the absence of one of these sensory cues, males are still able to court [6,23]. Our data confirmed that males with impaired vision (figure 1) or loss of olfaction (figure 2) or defect of gustation [17] could court females all the same, suggesting that vision, olfaction and gustation are not individually vital for male courtship behaviour. However, males with a defect in olfaction and gustation [17] failed to distinguish younger females from older ones, indicating that olfaction and gustation are necessary for males' courtship preference. Sex pheromones, which can be perceived by olfactory or gustatory sensory neurons, are involved in sexual behaviours among many insects, including *Drosophila* [25,30–32]. In *Drosophila*, both volatile and non-volatile pheromones play roles in courtship behaviour [9,25,33]. However, whether and how these pheromones function in male courtship preference are still unclear. Our previous data raised the possibility that non-volatile pheromones (cuticular hydrocarbons, CHCs) might contribute to male courtship preference through GR, Gr33a [17]. In this study, we demonstrated that Or47b was required for male courtship preference for younger mates. Another recently published work identified a female-specific volatile pheromone, methyl laurate (ML), which promotes male courtship behaviour, as a ligand of Or47b [10]. As a result, it is highly possible that ML, which acts as an attractive pheromone, and with higher concentration on younger than older females' bodies, plays a role in male courtship preference behaviour via Or47b. In order to verify this hypothesis, we made several attempts to measure the compounds on the bodies of younger and older females. However, with our current gas chromatography–mass spectrometry (GC-MS) equipment, we were unable to detect any robust profile of ML (data not shown). Most of the compounds detected were non-volatile CHCs, which is consistent with our previous data [17].

The wild life for *Drosophila* is tough; as a result, courtship behaviour, which promotes reproductive success, is not easily diminished by malfunction of merely a single sensory cue. However, courtship preference, which provides the possibility of optimized progenies of males, is much more sensitive to such sensory defects. In this sense, the males' courtship preference behaviour is so delicate and fragile that even tiny flaws in either olfactory or gustatory sensory inputs can completely abolish it. In summary, courtship preference, to some extent, is more advanced than courtship behaviour. In this study, we explored the function of vision and olfaction in *Drosophila* male courtship preference, and our findings revealed the critical role of Or47b in males' preference for younger mates, which expanded our knowledge of sensory inputs involved in such delicate behaviours.

## Supplementary Material

Supplementary figures and legends

## Data accessibility

The datasets supporting this article have been uploaded as part of the electronic supplementary material.
