# Supplementary material for: Or47b plays a role in Drosophila males' preference for younger mates
Source: Open Biol. 2016 Jun 8;6(6):160086. doi: 10.1098/rsob.160086 (PMC4929943; doi:10.1098/rsob.160086)
Supplement: Supplementary figures and legends [file rsob160086supp1.docx]

**Or47b Plays a Role in *Drosophila* Males’ preference for younger mates**

Luming Zhuang^1, 2^, Ying Sun^1, 2^, Mi Hu^3^, Chenxi Wu^1, 4^, Xiaojin La^4^, Xinhong Chen^1^, Yu Feng^1^, Xingjun Wang^1^, Yujia Hu^1, 5, *^ and Lei Xue^1, *^

^1^ Department of Interventional Radiology, Shanghai 10th People’s Hospital, Shanghai Key Laboratory of Signaling and Disease Research, School of Life Science and Technology, Tongji University, Shanghai 200092, China.

^2^ These authors contributed equally to this work.

^3^ Kent School, 1 Macedonia Rd, Kent, CT 06757, USA

^4^ College of Chinese Medicine, North China University of Science and Technology, Tangshan 063000, China

^5^ Present address: Life Sciences Institute, Department of Cell & Developmental Biology, University of Michigan, Ann Arbor, MI 48109, USA

^*^ Correspondence: [henryhu@umich.edu](mailto:henryhu415@hotmail.com) (Y.H.) or [lei.xue@tongji.edu.cn](mailto:lei.xue@tongji.edu.cn) (L.X.)

**Running title: Or47b Plays a Role in Preference**

**Keywords: Or47b, *Drosophila*, Courtship Preference**

**Supplementary Figures and Legends**


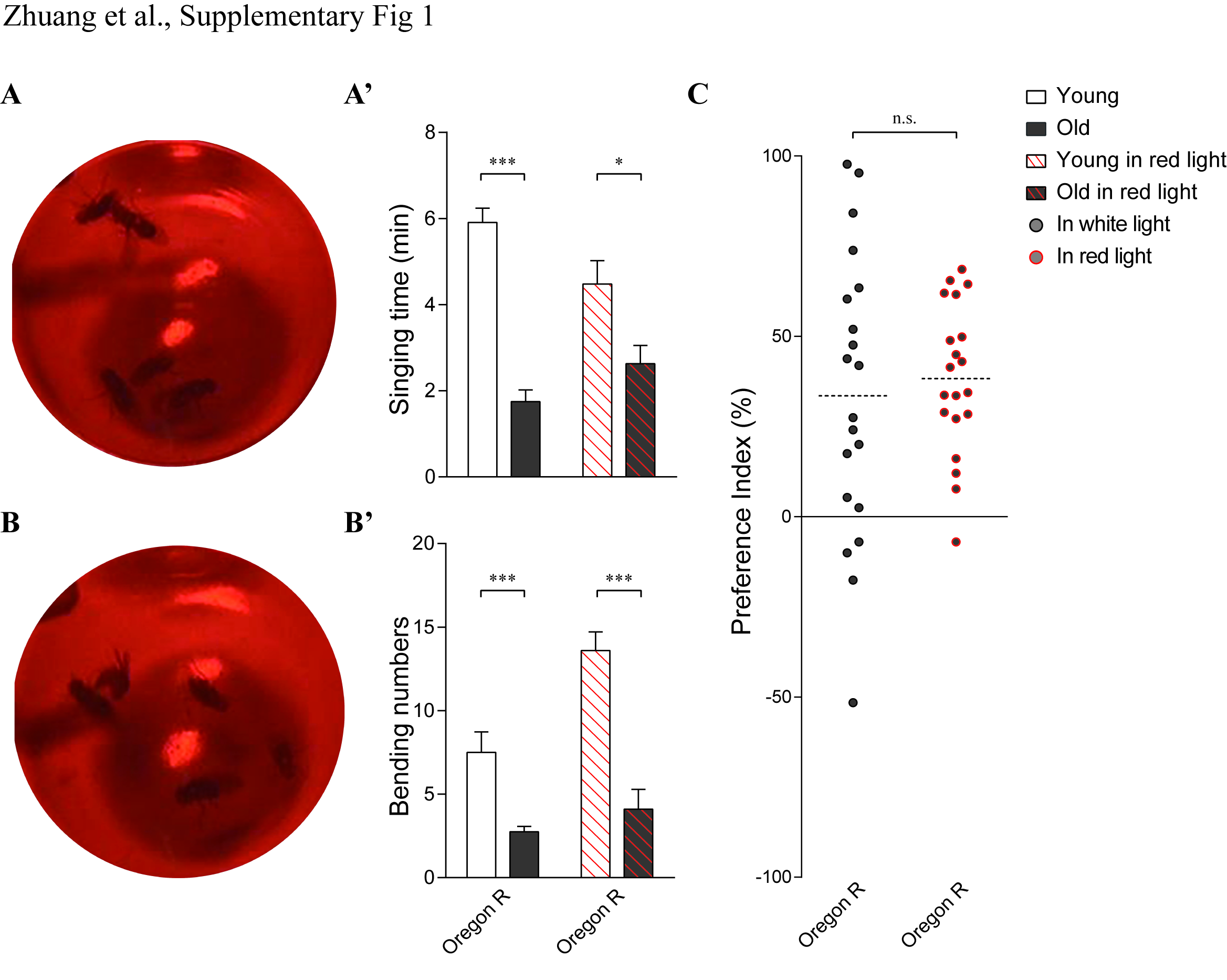


**Figure S1. Males still prefer younger mates under dim red light.**

**(A & A’)** Singing time of wild type males (*Oregon R*) under white light (solid bar) and dim red light (red slashed bar) respectively towards younger virgin females (white) and older ones (grey) in courtship choice assays. Asterisk, p < 0.05, three asterisks, p < 0.001, Related-samples Wilcoxon signed rank test. **(B & B’)** Bending numbers of wild type males under white light (solid bar) and dim red light (red slashed bar) respectively towards younger virgin females (white) and older ones (grey) in courtship choice assays. Three asterisks, p < 0.001, Related-samples Wilcoxon signed rank test. **(C)** Preference indices of wild type males under white light (solid bar) and dim red light (red slashed bar) in courtship choice assays. n.s., p > 0.05, Mann-Whitney *U* test.


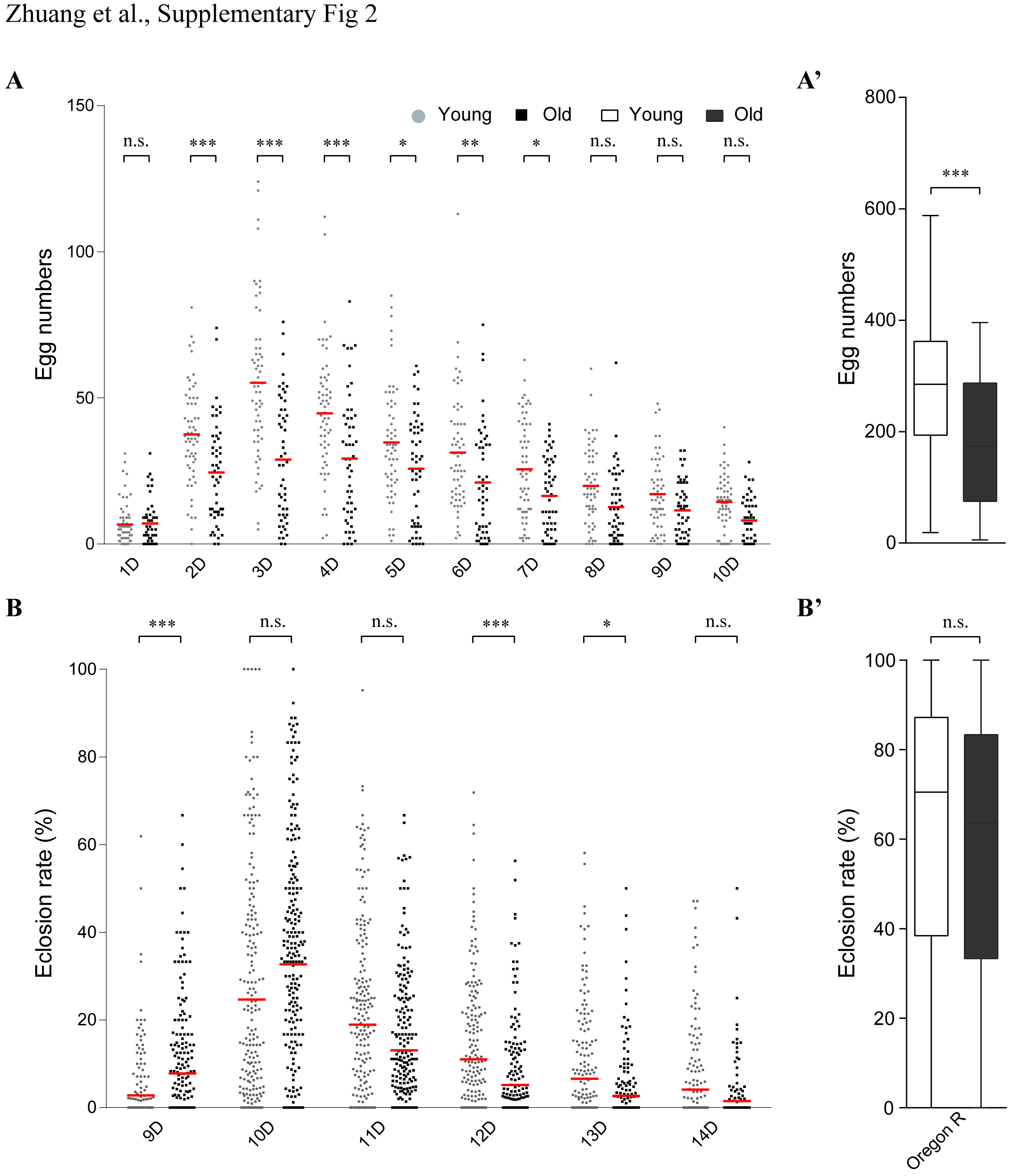


**Figure S2. Younger females have better reproductive capacity than older ones.**

**(A)** The number of eggs laid by younger wild type (*Oregon R*) females (white) or older ones (grey) each day after copulation. Asterisk, p < 0.05, two asterisks, p < 0.01, three asterisks, p < 0.001, two-way ANOVA followed by Sidak's multiple comparisons test. **(A’)** The total number of eggs laid by younger wild type females (white) or older ones (grey) during 10 days after copulation. Three asterisks, p < 0.001, independent student t test. **(B)** The eclosion rate of eggs laid by younger wild type females (light grey) or older ones (grey) from 9th to 14th day after copulation. Asterisk, p < 0.05, three asterisks, p < 0.001, Kruskal-Wallis test, Dunn’s post-hoc. **(B’)** The total eclosion rate of eggs laid by younger females (white) or older ones (grey). n.s., p > 0.05, Mann Whitney *U* test.


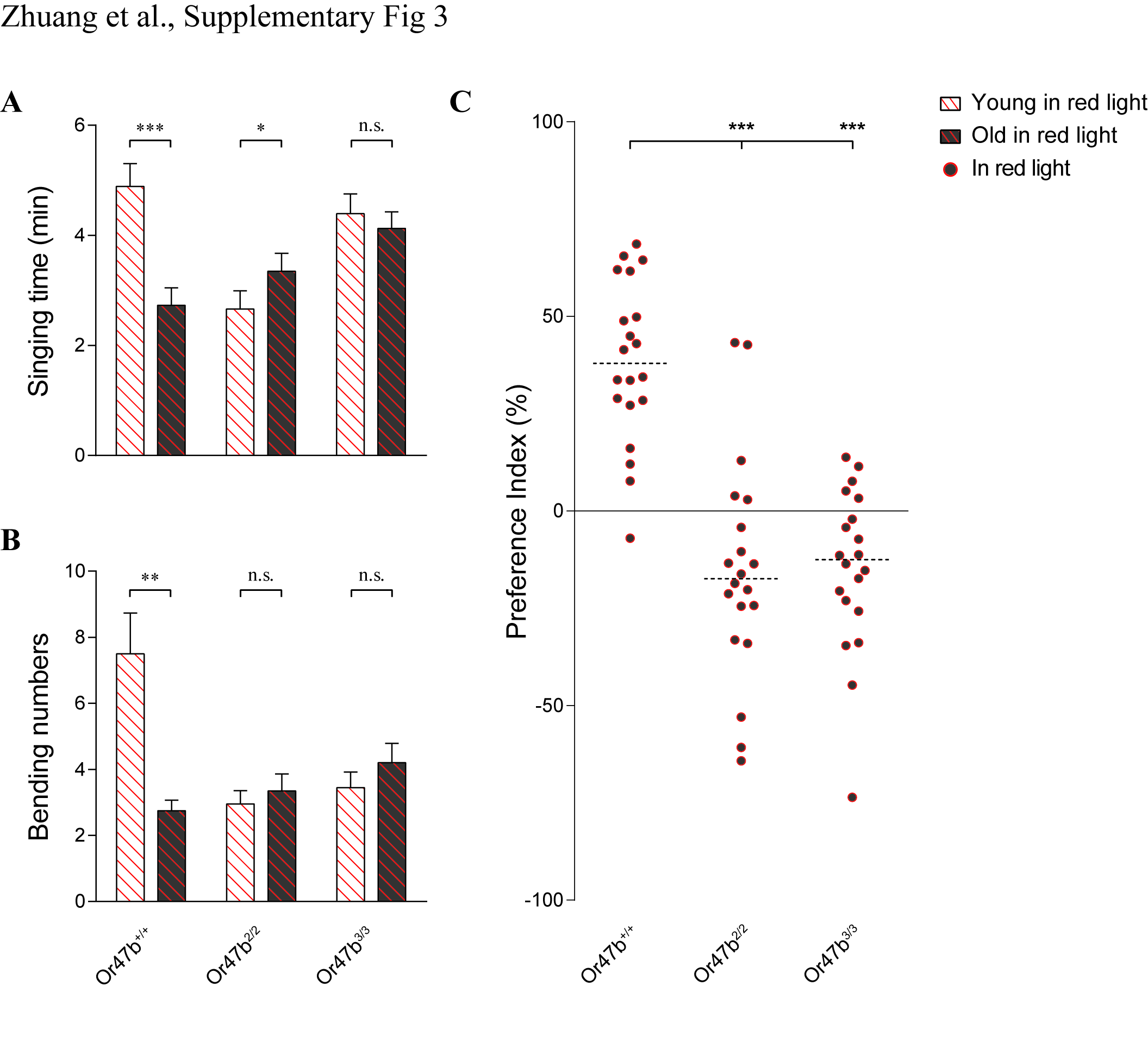


**Figure S3. Or47b is still required for males’ preference behavior** **under dim red light.**

**(A)** Singing time of control males (*Or47b*^+/+^) and *Or47b* mutant males (*Or47b^2/2^* and *Or47b^3/3^*) towards younger females (white with red slash) and older ones (grey with red slash) under dim red light in courtship choice assays. n.s., p > 0.05, asterisk, p < 0.05, three asterisks, p < 0.001, Related-samples Wilcoxon signed rank test. **(B)** Bending numbers of control males and mutant males towards younger females (white with red slash) and older ones (grey with red slash) under dim red light in courtship choice assays. n.s., p > 0.05, two asterisks, p < 0.01, Related-samples Wilcoxon signed rank test. **(C)** Preference indices of control males and mutant males in courtship choice assays under dim red light. Three asterisks, p < 0.001, Kruskal-Wallis test, Dunn’s post-hoc.


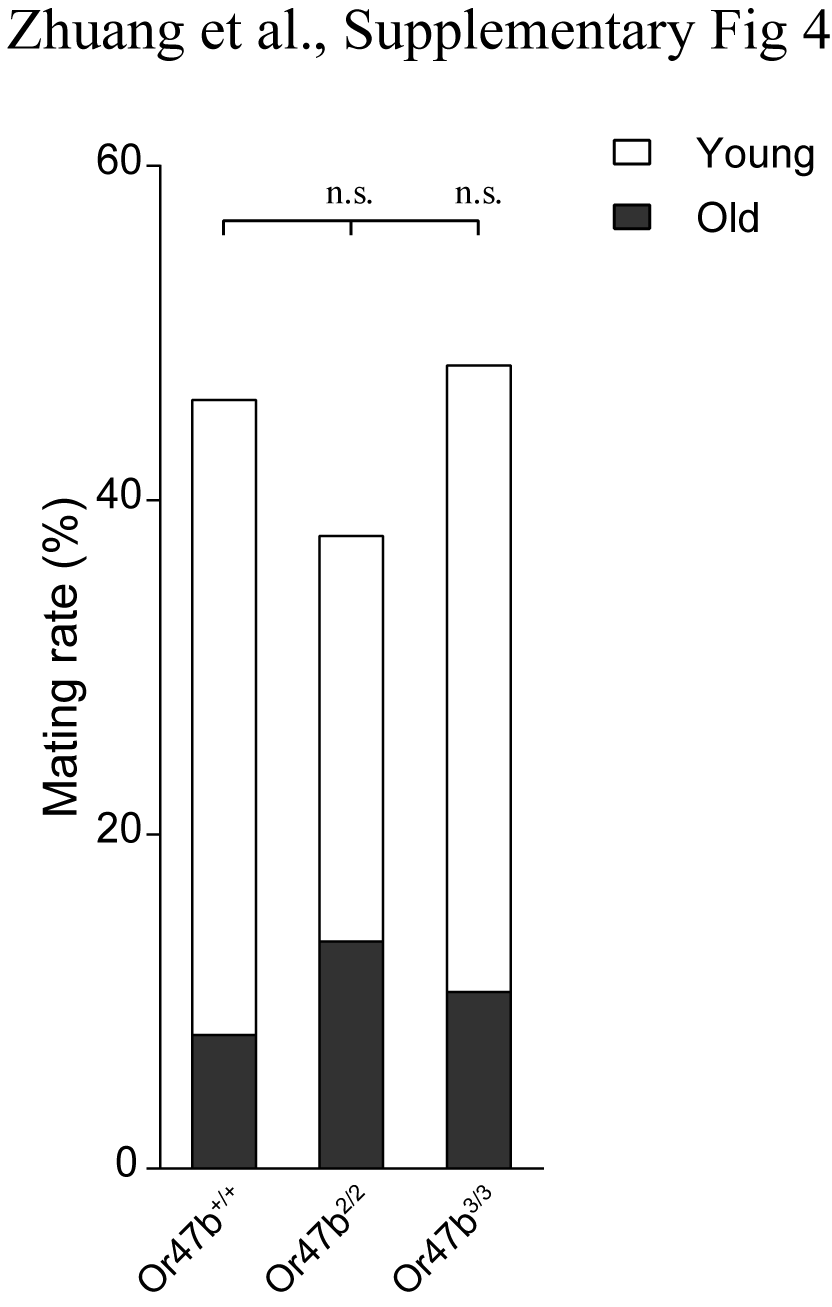


**Figure S4. Male courtship preference was not necessarily the indicator of copulation success.**

The mating rates of control males (*Or47b*^+/+^) and mutant males (*Or47b^2/2^* and *Or47b^3/3^*) with younger females (white) or older ones (grey) in courtship choice assays within 30 minutes. n.s., p > 0.05, Pearson's Chi-squared test with Yates' continuity correction.


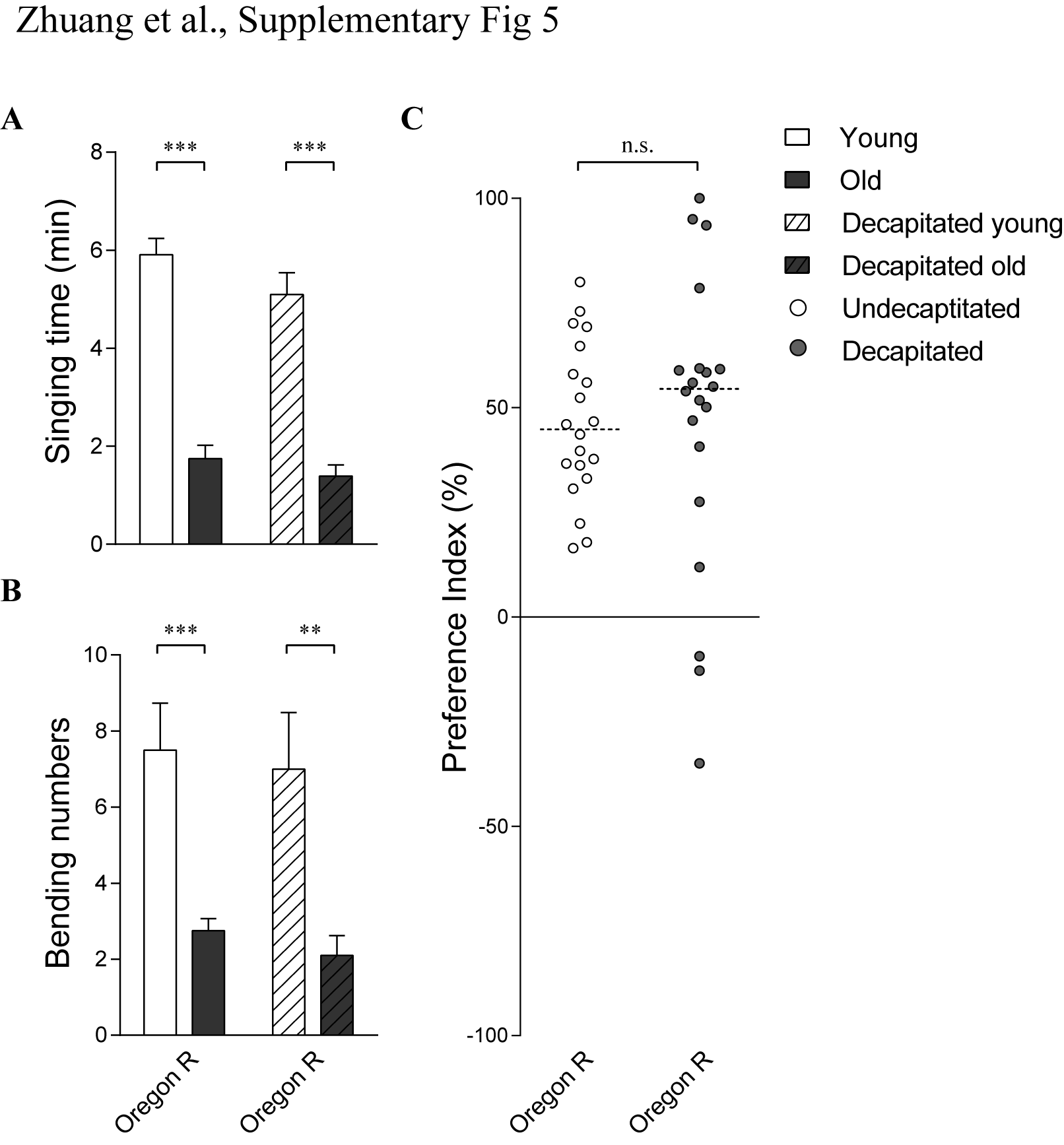


**Figure S5. Males’ courtship preference is not affected by females’ choice.**

**(A)** Singing time of wild type males (*Oregon R*) towards intact (white bar) or decapitated (white with slash) younger virgin females, and intact (grey) or decapitated (grey with slash) older ones in courtship choice assays. Mean ± standard error of the mean (SEM). n.s., p > 0.05, three asterisks, p < 0.001, Related-samples Wilcoxon Signed Rank test. **(B)** Bending numbers of wild type males towards intact (white) or decapitated (white with slash) younger virgin females, and intact (grey) or decapitated (grey with slash) older ones in courtship choice assays. Mean ± standard error of the mean (SEM). Three asterisks, p < 0.001, two asterisks, p < 0.01, Related-samples Wilcoxon Signed Rank test. **(C)** Preference indices of wild type males towards intact or decapitated females in courtship choice assays. Scatter dot plot with dotted line at median. n.s., p > 0.05, Mann Whitney *U* test.
